# Karyotypic diversity in the Gobiidae: A comparative analysis of two Atlantic species

**DOI:** 10.3897/compcytogen.20.206266

**Published:** 2026-08-12

**Authors:** Paulo Augusto de Lima-Filho, Gideão Wagner Werneck Félix da Costa, Simião Alefe Soares da Silva, Wagner Franco Molina

**Affiliations:** 1 Instituto Federal de Educação, Ciência e Tecnologia do Rio Grande do Norte, Santa Cruz – RN, Brazil Instituto Federal de Educação, Ciência e Tecnologia do Rio Grande do Norte Santa Cruz Brazil; 2 Departamento de Biologia Celular e Genética, Centro de Biociências, Universidade Federal do Rio Grande do Norte, Natal – RN, Brazil Departamento de Biologia Celular e Genética, Centro de Biociências, Universidade Federal do Rio Grande do Norte Natal Brazil

**Keywords:** Atlantic fishes, Chromosomal rearrangements, fish cytogenetics, karyotype evolution

## Abstract

Gobies (family Gobiidae) rank among the most diverse of all marine fish families. Although the cytogenetics of these small, ubiquitous fish is still a young field, studies on Atlantic species, despite their limited number, have already revealed a remarkable level of karyotypic variation. This karyotype diversity has provided critical evidence for discovering new species, uncovering population structures, identifying sex chromosomes, and pinpointing major shifts in repetitive DNA. This study presents the first chromosomal characterization of two Atlantic goby species, *Coryphopterus glaucofraenum* (Gill, 1863) and *Evorthodus lyricus* (Girard, 1858), using classical cytogenetics and base-specific fluorochrome staining. Our analyses revealed a striking divergence in their karyotypes. *Evorthodus lyricus* exhibited a karyotype of 2n = 46 (FN = 92), whereas *C. glaucofraenum* presented 2n = 40 (FN = 42), indicating significant differences in both chromosome and fundamental number (FN). These cytogenetic patterns, combined with existing data for the family, reinforce the high karyotypic diversity within Gobiidae and suggest a relationship between speciation processes and chromosomal modifications in the diversification of this family.

## Introduction

Gobiidae are the most diverse family in the marine environment, with 1,440 recognized species (Fricke et al. 2025). Their representatives are distributed across tropical, subtropical, and temperate regions in both coastal and freshwater habitats (Nelson et al. 2016), and they form an important and predominant component of the fish fauna in reef environments (Greenfield and Johnson 1999). However, due to their multiplicity of habitats, wide geographic distribution, small body size, and often cryptic ecological patterns (Taylor and van Tassell 2002), the full extent of this group’s diversity (Thacker and Roje 2011; Hammer et al. 2021) remains largely unknown.

The remarkable diversity in Gobiidae appears to have been accompanied by significant chromosomal changes, diverging from the basal Percomorpha karyotype of 48 acrocentric chromosomes (Motta-Neto et al. 2019), a pattern more common in marine than freshwater species (Brum 1995; Brum and Galetti Jr 1997; Galetti-Jr et al. 2000). Indeed, cytogenetic data for gobies indicate a derived karyotypic pattern in many species (Galvão et al. 2011; Silva et al. 2021), with karyotypes ranging from 2n = 30 in *Neogobius euricephalus* (Ene, 2003) to 2n = 56 in *Gobionellus microdon* (Uribe-Alcocer and Diaz-Jaimes 1996). Frequent and complex modifications in the karyotypic structure of gobies appear to be linked to the evolutionary pattern of this group (Caputo et al. 1998).

In an effort to advance the understanding of karyotype evolution in Gobiidae, this study provides a cytogenetic analysis of *Coryphopterus glaucofraenum* (Gill, 1863), a reef-dwelling species, and *Evorthodus lyricus* (Girard, 1858), a marine and estuarine species, both of which exhibit a broad geographic distribution from the USA to southern Brazil (Menezes et al. 2003).

## Material and methods

Individuals of *E. lyricus* (N = 9; 4♂/5♀) and *C. glaucofraenum* (N = 7; 4♂/3♀) were collected from the coast of Rio Grande do Norte, in the Northeast region of Brazil. The individuals were subjected to *in vivo* mitotic stimulation overnight via intramuscular inoculation of a complex of bacterial and fungal antigens (Molina et al. 2010). Mitotic chromosomes were obtained from a cell suspension of the anterior kidney following short-term culture (Gold et al. 1990). The diploid number was established by analyzing approximately thirty metaphase spreads per individual, stained with 5% Giemsa diluted in phosphate buffer (pH 6.8). Heterochromatic regions were analyzed using C-banding (Sumner 1972), and the major ribosomal sites of the nucleolar organizer regions (NORs) were identified using the Ag-NOR technique (Howell and Black 1980). Chromosomes were sequentially stained with the base-specific fluorochromes CMA_3_ and DAPI (Carvalho et al. 2005). The best metaphase spreads were photographed using an Olympus™ BX51 microscope equipped with an Olympus DP73 digital capture system and used for karyotype assembly. Chromosome morphology was determined according to Levan et al. (1964). All individuals’ collection and handling procedures were approved by the Ethics Committee of the Federal University of Rio Grande do Norte (UFRN; Protocol 44/2015). Additionally, fieldwork and sampling were conducted under collection licenses provided by the Instituto Brasileiro do Meio Ambiente e dos Recursos Naturais Renováveis (IBAMA; Processes #19135-8, #131360-1, and #27027-2).

## Results

**Figure 1.**
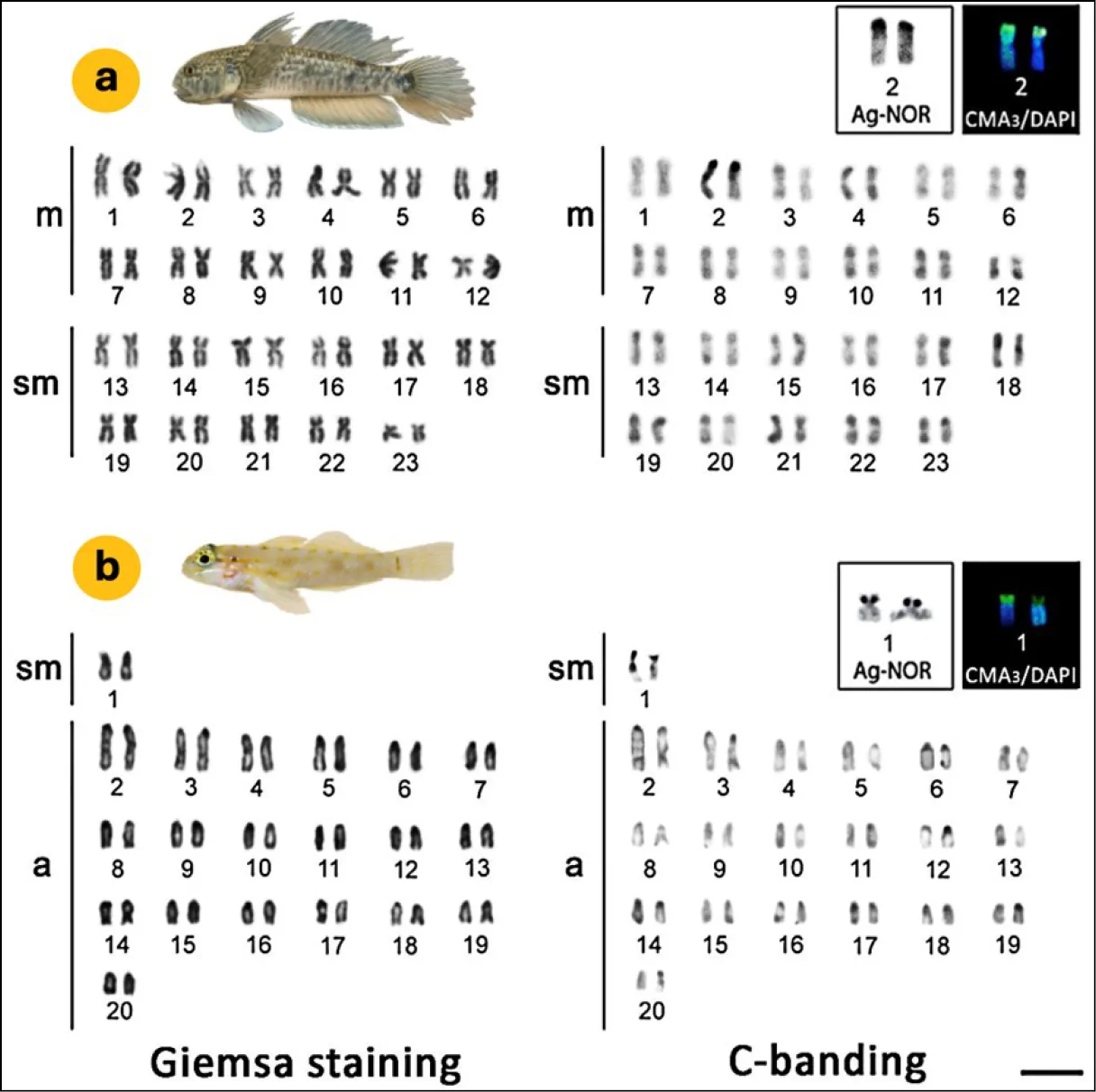
Karyotypes *Evorthodus lyricus* (a, ♂ and ♀) and *Coryphopterus glaucofraenum* (b, ♀ and ♂) after Giemsa staining and C-banding. No karyotypic differences were observed between the sexes. The boxes highlight the nucleolus organizer pair, identified by Ag-NORs staining and sequentially confirmed to be CMA_3_^+^/DAPI^−^. Scale bar: 5 µm.

The cytogenetic analyses carried out on *E. lyricus* and *C. glaucofraenum* revealed entirely differentiated 2n and karyotypic macrostructures. While *E. lyricus* exhibited 2n = 46 (22m+24sm, FN = 92), *C. glaucofraenum* exhibited 2n = 40 (2sm+38a, FN = 42) (Fig. 1). In the latter species, the numerical reduction in the diploid number is accompanied by the presence of acrocentric pairs that are significantly larger than the other chromosomes in the karyotype. No cytogenetic differences or sex-linked chromosome heteromorphisms were observed between male and female specimens in either of the species analyzed.

In both species, the Ag-NOR sites are located in the terminal position on the short arm of a two-armed chromosome pair, identified as pair 2 (metacentric) in *E. lyricus* and pair 1 (submetacentric) in *C. glaucofraenum*. These sites are heterochromatic and represent the only chromosomal regions that exhibit a CMA_3_^+^/DAPI^−^ staining pattern in the karyotypes (Fig. 1; boxes). Heterochromatic regions were conspicuous and terminally distributed on most chromosomes in *E. lyricus*, while in *C. glaucofraenum*, they were reduced and primarily located in centromeric regions.

## Discussion

The karyotypic patterns of *C. glaucofraenum* (2n = 40; 2sm+38a) and *E. lyricus* (2n = 46; 22m+24sm) corroborate the high numerical and structural karyotypic diversity previously indicated for the Gobiidae family (Silva et al. 2021).

*Evorthodus lyricus* exhibited 2n = 46 chromosomes, which is considered the basal diploid number for the family (Silva et al. 2021). However, the extensive presence of two-armed chromosomes (FN = 92) indicates the intense action of chromosomal rearrangements in establishing this karyotypic pattern. Although *C. glaucofraenum* has 2n = 40, a 2n lower than the considered basal for the family, its first acrocentric pairs are disproportionately larger than the others. This suggests that *in tandem* fusion acted as the primary rearrangement, a common mechanism observed in the karyotypic diversification of other gobiids, such as *Gobius paganellus* (Amores et al. 1990). Because conventional cytogenetic protocols do not provide the resolution needed to map precise structural breakpoints, the exact pathways of these rearrangements (such as pericentric inversions vs. centromere repositioning, or tandem vs. centric fusions) remain to be tested using high-resolution molecular cytogenetics.

The location and frequency of Ag-NOR sites, which constitute consistent cytotaxonomic markers for many fish groups (Caputo et al. 1998), are highly variable in gobiids (Ene 2003) and have revealed unique patterns useful for the identification of cryptic species (Lima-Filho et al. 2012). In *E. lyricus* and *C. glaucofraenum*, the Ag-NOR sites are single, a condition considered symplesiomorphic not only for teleosts but also for most vertebrates (Amemiya and Gold 1986). In both species, the ribosomal sites are heterochromatic and GC-rich, a condition found in the nucleolar organizer pairs of various species within the family (Mandrioli et al. 2001; Ene 2003; Lima-Filho et al. 2012, 2014).

Heterogeneous heterochromatic regions, rich in various types of repetitive sequences, including 5S rDNA sites, have been suggested to facilitate chromosomal fusion events in some marine Percomorpha species (Molina and Galetti-Jr 2002; Araújo et al. 2010; Getlekha et al. 2016). Among Gobiidae, evidence of heterochromatic heterogeneity has been identified in several species, such as *Gobius paganellus*, *G. niger*, *G. cobitis*, and *Zosterisessor ophiocephalus* (Caputo et al. 1997), and *Bathygobius soporator* (Lima-Filho et al. 2012). This heterogeneity has been implicated in the occurrence and fixation of chromosomal modifications, including events of centric fusions and fissions (Canapa et al. 2002). Furthermore, while heterochromatin dynamics have also been linked to the emergence of heteromorphic sex chromosomes in Gobiidae (Lima-Filho et al. 2014), our cytogenetic data did not reveal any sex-specific chromosome differences in either of the species analyzed.

A karyotype with 2n = 46 acrocentric chromosomes has been considered basal for the family Gobiidae (Silva et al. 2021). From this ancestral state, variations in diploid number and the presence of two-armed chromosomes represent more derived conditions (Vasil’ev and Grigoryan 1993). A total of 84% of cytogenetically described species (Arai 2011; Silva et al. 2021) diverge from this karyotypic pattern, suggesting a close relationship between diversification processes and chromosomal changes within the family.

The majority of available cytogenetic data for gobies, largely limited to diploid numbers and karyotypic macrostructure (Caputo et al. 1998), still comes from initial studies on Mediterranean and Pacific species (e.g., Arai and Sawada 1974; Vitturi and Catalano 1989; Klinkhardt 1992; Caputo et al. 1997). Our results from Atlantic species reinforce the high karyotypic diversity within this group and underscore the close relationship between phyletic diversification processes and chromosomal changes in the Gobiidae family. These karyotype modifications have the potential to alter recombination frequencies and lead to the formation of chromosomally incompatible subpopulations, a process that can drive speciation.

## Additional information

### Artificial Intelligence (AI) use

The authors accept full responsibility for the content of the manuscript, including the disclosure of any use of AI.

No AI tools were used in the preparation of this manuscript.

### Author ORCIDs

P.A. de Lima-Filho https://orcid.org/0000-0002-9458-6860

G.W.W.F. da Costa https://orcid.org/0000-0001-7899-2677

S.A.S. da Silva https://orcid.org/0009-0005-6503-3174

W.F. Molina https://orcid.org/0000-0002-6695-0952

### Data availability

All of the data that support the findings of this study are available in the main text.
